# Generation of recombinant baculovirus expressing atoxic C-terminal CPA toxin of *Clostridium perfringens* and production of specific antibodies

**DOI:** 10.1186/s12896-019-0597-4

**Published:** 2020-01-28

**Authors:** Katia Forti, Monica Cagiola, Martina Pellegrini, Lucia Anzalone, Antonella Di Paolo, Sara Corneli, Giulio Severi, Antonio De Giuseppe

**Affiliations:** 1 Istituto Zooprofilattico Sperimentale dell’Umbria e delle Marche “Togo Rosati”, Via G. Salvemini 1, 06126 Perugia, Italy; 2 Dipartimento di Medicina Veterinaria, Università degli Studi di Perugia, Via San Costanzo 4, 06126 Perugia, Italy

**Keywords:** *Clostridium perfringens*, *L21* leader sequence, Atoxic rBacCPA250–363H6, Affinity chromatography, Recombinant vaccines

## Abstract

**Background:**

*Clostridium perfringens* is the causative agent of several diseases and enteric infections in animals and humans. The virulence of *C. perfringens* is largely attributable to the production of numerous toxins; of these, the alpha toxin (CPA) plays a crucial role in histotoxic infections (gas gangrene). CPA toxin consists of two domains, i.e., the phospholipase C active site, which lies in the N-terminal domain amino acid (aa residues 1–250), and the C-terminal region (aa residues 251–370), which is responsible for the interaction of the toxin with membrane phospholipids in the presence of calcium ions. All currently produced clostridial vaccines contain toxoids derived from culture supernatants that are inactivated, mostly using formalin. The CPA is an immunogenic antigen; recently, it has been shown that mice that were immunized with the C-terminal domain of the toxin produced in *E. coli* were protected against *C. perfringens* infections and the anti-sera produced were able to inhibit the CPA activity. Monoclonal and polyclonal antibodies were produced only against full-length CPA and not against the truncated forms.

**Results:**

In the present study, we have reported for the first time; about the generation of a recombinant baculovirus capable of producing a deleted rCPA toxin (rBacCPA250–363H6) lacking the N-terminal domain and the 28 amino acids (aa) of the putative signal sequence. The insertion of the *L21* consensus sequence upstream of the translational start codon ATG, drastically increases the yield of recombinant protein in the baculovirus-based expression system. The protein was purified by Ni-NTA affinity chromatography and the lack of toxicity in vitro was confirmed in CaCo-2 cells. Polyclonal antibodies and eight hybridoma-secreting Monoclonal antibodies were generated and tested to assess specificity and reactivity. The anti-sera obtained against the fragment rBacCPA250–363H6 neutralized the phospholipase C activity of full-length PLC.

**Conclusions:**

The *L21* leader sequence enhanced the expression of atoxic C-terminal recombinant CPA protein produced in insect cells. The monoclonal and polyclonal antibodies obtained were specific and highly reactive. The availability of these biologicals could contribute to the development of diagnostic assays and/or new recombinant protein vaccines.

## Background

*Clostridium perfringens* is an anaerobic, spore-forming bacterium that is widely distributed in the environment and is a part of the normal microbiota flora of the gastrointestinal tract of humans and animals. However, this ubiquitous, gram-positive, saprophyte, in certain circumstances, causes many enterotoxemic diseases and different forms of tissue damage (lamb dysentery, gas gangrene, food poisoning, and necrotic enteritis). Though *C. perfringens* does not invade healthy cells, it produces a wide range of potent extracellular toxins and enzymes that are responsible for the associated lesions and symptoms. Toxin production, which varies significantly among *C. perfringens* strains, is the basis for a classification system that, has been recently revised to include seven toxinotypes (A, B, C, D, E, F, G), based upon the presence of genes encoding for alpha (CPA or PLC), beta (CPB), epsilon (ETX) and iota (ITX) toxins, and enterotoxin (CPE) and necrotic enteritis B-like toxin (NetB) [1]. However, this microorganism can produce at least 17 toxins in various combinations, including lethal toxins, such as perfringolysin O (PFO) and beta2 toxin (CPB2).

CPA is the most important virulence factor involved in human clostridial myonecrosis [2, 3] or histotoxic infections such as those causing gas gangrene [4, 5]. The CPA encoding gene (*cpa* or *plc*) is situated in a stable region within the bacterial chromosome and CPA is produced by all *C. perfringens* strains, although the amounts produced by toxinotype A strains are usually higher than those produced by other toxinotypes [6]. The VirS/VirR-VR-RNA signal transduction cascade (QS systems) regulating the production of the toxin genes chromosomally located as *cpa*, *pfoA* and *colA* genes [7, 8]. Recently studies stated that a down regulation of the QS regulatory systems is mediated by primary acidic metabolites and acidic environments, suggesting the possibility of pH-controlled anti-virulence strategies [9]. Alpha toxin is a 43 kDa metallic enzyme comprised of 370 amino acids, which is secreted due to the presence of a signal peptide [10–12]. Three-dimensional analysis revealed that it consists of two domains, i.e., the catalytic α-helical N-terminal zinc-binding domain (aa residues 1–250) that exhibited phospholipase C (PLC) and sphingomyelinase (SMase) activities, and the antiparallel β-sandwich C-terminal calcium-binding region (aa residues 251–370), that influences the enzymatic activity of the N-terminal domain and is involved in the interaction between the toxin and membrane phospholipids [6, 13, 14]. CPA also has two flexible loops (central domain 55–93 aa and 132–149 aa) and its first domain contains a ganglioside (GM1a) binding site [14, 15].

Jepson et al. studied the difference between the C- terminal domains of *C. perfringens* and *C. bifermentans* and confirmed that the C-terminal domains of these proteins conferred different properties on the enzymatically active N-terminal domains of these proteins [16].

Both domains are immunogenic, but only the C-terminal domain stimulates a protective immune response [17, 18]. To determine which components of the C-terminal domain of the alpha-toxin were the strongest immunogens, Nagahama et al. immunized mice with various recombinant fragments produced in *E. coli* and evaluated their capacity to protect against *C. perfringens* infections [18]. Moreover, Goossens et al. demonstrated the protection afforded by antisera raised against the C-terminal domain of CPA toxin, concluding that the recombinant C-terminal CPA could replace the full length CPA as a vaccine component [19]. The role of CPA in causing intestinal disease in mammals is controversial and poorly documented. In fact, though large amounts of CPA are detected in the faeces of naturally infected cattle, CPA is also present in the intestinal content of many clinically healthy animals [6]. The activity of CPA is highly complex, and it varies among cell types due to factors such as the ratio of phosphatidylcholine (PC) to sphingomyelin (SM) in the plasma membrane, and it is also influenced by toxin concentrations. This toxin was previously considered to act only locally on the cell membrane, but it has recently been shown that CPA has a cytotoxic effect at sublytic concentrations if it has been endocytosed and trafficked through the endolysosomal route [20]. Moreover, Takagishi et al. reported that the CPA toxin disturbed the innate immune system, by impairing granulopoiesis, inhibiting neutrophil differentiation, and decreasing the number of mature erythroblasts [21]. Therefore, despite the fact that CPA is the most studied *C. perfringens* toxin and the first bacterial toxin that had been shown to have an enzymatic activity [22], there are still some gaps in our knowledge regarding its molecular mechanism of action and its role as a component of the microbiota and in gastrointestinal diseases in humans and animals. Moreover, other interesting aspects include the antigenic architecture of CPA, availability of efficient diagnostic tools, and evaluation of its use in the production of a new generation of vaccines. Commercial diagnostic tests to detect the CPA toxin are available, but they are all very expensive and based on antibodies directed against the full-length 370 aa protein, and not against the truncated C-terminal domain of the toxin that was produced only in prokaryotic systems.

The aim of the present study was to generate, for the first time, a recombinant baculovirus capable of producing an N-terminal truncated CPA protein of *C. perfringens*. Previous authors reported that a *cis* element of 21 bp (*L21*) derived from a 5′ untranslated leader sequence of a lobster tropomyosin cDNA, dramatically increased the expression levels of exogenous genes in baculovirus-infected insect cells [23]. *L21* (AACTCCTAAAAAACCGCCACC) has both the vertebrate *Kozak* sequence (CCGCCACC) and the A-rich sequence (TAAAAAA) found in the polyhedrin leader sequence, and it is well known that the A-rich sequence increased the expression level in Sf9 cells [23]. In order to improve the translation efficiency of the baculovirus-based expression system, two different *cis* regulatory elements, a *L21* consensus sequences and a selected *Kozak* sequence previously used in other studies [24] were inserted and the resultant levels of protein expression achieved were compared in this study. Monoclonal and polyclonal antibodies, that were obtained and directed against the purified non-toxic C-terminal rBacCPA250–363H6 recombinant protein, were tested to evaluate their reactivity and specificity in immunoblotting assays and their eventual use in *home-made* immunoenzymatic assays, which were able to detect native CPA from field strains of *C. perfringens*.

## Methods

### Preparation of *C. perfringens* culture supernatant

Broth culture supernatants of different *C. perfringens* toxinotypes were collected and evaluated to assess the levels of cytotoxicity and phospholipase C activity. The toxinotype of *C. perfringens* strains was determined by a colony multiplex polymerase chain reaction [25]. Bacteria were isolated and grown anaerobically on blood agar overnight at 37 °C. Then, a single colony of each isolate was inoculated into 5 mL of TGY broth (3% tryptone, 2% yeast extract, 0.1% glucose, and 0.1% L-cysteine) and grown anaerobically at 37 °C for 24-48 h. The broth cultures were then centrifuged filtered (0.22 μm) and stored at − 20 °C for future use.

### Cloning of gene encoding N-terminal deleted rBacCPAH6 plasmids expressing in insect cell

Bacterial genomic DNA was isolated from the culture supernatant of *C. perfringens* ATCC 13124, which contained the chromosomal gene *cpa* [26], using a QIAamp®DNA Mini Kit (QIAGEN, GmbH, Germany) and stored at − 20 °C until it was used as template to amplify the deleted recombinant forms of rBacCPAH6. Based on the DNA sequence of the CPA toxin in GenBank [26, 27], specific primer sets were designed (Table 1).

**Table 1:** Primer sequences of the recombinant truncated rBacCPAH6 proteins. The restriction sites and the start codons are underlined with a solid line, in bold the consensus leader sequences

Primers	Nucleotide sequences
rBacCPA 279- *Eco*RI^b^-F	5’-AAGAGAATTC^b^TGTATATG^c^GGAATCAAAACAAAGGATGT-3’
rBacCPA 279***Kozak***^a^-*Eco*RI^b^-F	5’-AAGAGAATTC^b^**CAAA**^a^ATG^c^GGAATCAAAACAAAGGATGT-3’
rBacCPA 279***L21***^a^-*Eco*RI^b^-F	5’-TGATGAATTC^b^**AACTCCTAAAAAACCGCCACC**^a^ATG^c^GGAATCAAAACAAAGGA-3’
rBacCPA250***L21***^a^-*Eco*RI^b^-F	5’-ATTCGAATTC^b^**AACTCCTAAAAAACCGCCACC**^a^ATG^c^TCAGTTGGAAAGAATG -3’
rBacCPA 363-*Age*I^b^- R	5’-AAGTACCGGT^b^TCCTGAAATCCACTCGTTTATATC-3’
rBacCPA 370-*Age*I^b^- R	5’-ACTTACCGGT^b^TTTTATATTATAAGTTGAATTTCCTGAAATC-3’

^a^in bold= consensus leader sequences

^b^with underline= restriction site

^c^with underline= start codon

In order to increase recombinant protein expression levels and evaluate the importance of consensus leader sequences (*Kozak* and *L21*), three types of forward primers were designed that differed with regard to the nucleotide sequences present immediately upstream of the translational start codon ATG. In the first forward primer, no consensus sequence was present, while in the second and third forward primers, consensus *Kozak* and *L21* sequences were inserted, respectively. Essentially, DNA fragments encoding the domain CPA279–363, was amplified from the previously extracted genomic DNA by PCR, using a Taq Polymerase AccuPrimePfx with high fidelity (Promega) and primers that caused the addition of *Eco*RI and *Age*I sites at the 5′ and 3′-ends of the product, respectively.

The constructs encoding the domains CPA279–370, CPA250–363 and CPA250–370 were generated as previously described bearing only the *L21* sequence.

The amplified products were analysed by 1% agarose gel electrophoresis, purified using the QIAquick PCR purification kit (QIAGEN), and cloned in the pOET2C-6xHis transfer vector *in frame* with the 6xHis-tag, using appropriate restriction sites (*Eco*RI and *Age*I). The resultant plasmid constructs were confirmed by sequence analysis. The recombinant baculoviruses expressing all the truncated rBacCPAH6 proteins were obtained by co-transfection of Sf21 insect cells with the constructs and with *flash*BAC DNA, as reported in the manufacturer’s instructions of the *flash*BAC system (Oxford Expression Technologies, UK). Recombinant viruses titer (IFU/ml) were determined using the BacPAK Baculovirus Rapid Titer Kit (Clontech Laboratories, Inc).

### Expression of deleted rBacCPAH6 proteins

The *Spodoptera frugiperda* insect cell line Sf21 (Thermo Fisher) was cultured in three different insect mediums, two serum-free mediums, HYQ SFX (HyClone), EX-cell 420 (Sigma) and Grace’s medium (Thermo Fisher) containing 10% Foetal Bovine Serum (FBS) in cell culture flasks at 27 °C. The large-scale production of the recombinant proteins was performed after infecting Sf21 cells in serum free EX-cell 420 medium with the recombinant baculoviruses, according to the manufacturer’s instructions. For estimate the expression level of protein with the different consensus leader sequences (*L21* and *Kozak*) 7.5 × 10^6^ Sf21 cells were infected in 10 ml of an EX-Cell 420 serum free medium with recombinant virus at a multiplicity of infection (MOI) of 2.0. After 72 h post-infection the infected cells were harvested and stored at − 20 °C until further use.

### SDS-PAGE, Coomassie and Western blotting

Protein levels were compared by lysing the infected and collected Sf21 cells in lysis buffer (0.1 M sodium phosphate buffer at pH 8, 8 M urea, 4 mM DTT) in ice for 1 h. After centrifugation at 16,000×g for 20 min at 4 °C the soluble fractions were quantified by Bradford method and an equal amount of all samples were mixed with 4xNuPage sample buffer containing 100 mM DTT and heated at 95 °C for 10 min. The denatured proteins were separated on a 12% SDS-PAGE precasting gel that was run for 1 h at 200 V and then staining with Bio-SafeCoomassie® G-250 (Simply Blue SafeStain, Thermo Fisher Scientific) or electroblotted onto a PVDF membrane. The membranes were blocked for 3 h at room temperature with TBST buffer (20 mM Tris, 150 mM NaCl pH 7.4 plus 0.05% (v/v) Tween 20) containing 5% (w/v) skimmed milk. After incubating the membranes overnight (ON) with anti-His6-HRP conjugated MAbs, signals were detected using the SuperSignal West Pico Chemiluminescence kit (Thermo Fisher Scientific) with Alliance Mini HD9Western Blot Imaging System (Eppendorf).

### Purification of his-tagged deleted recombinant rBacCPAH6 proteins

Proteins produced from baculovirus infected Sf21 cells were purified using a Ni-NTA His-select Nickel Affinity Gel (SIGMA-Aldrich) in denaturing conditions, according to the protocol provided by the manufacturer. The truncated rBacCPAH6 proteins that lacked the *putative* signal sequence encoding the first 28 amino acids were localized in the cell fraction. The cells were harvested by centrifugation and treated with the lysis buffer as previously described. Protein soluble fractions were recovered after the centrifugation of cellular lysates at 16,000×g for 20 min at 4 °C after performing Ni-NTA. The purified proteins were eluted in buffers with a pH of 5 and 4.5, according to the manufacturer’s instructions, and dialysed overnight in PBS buffer with 2 K MWCO slide–A-lyzer cassette G (Thermo Scientific). The protein concentration was determined using the Bradford protein assay reagent (Bio-Rad) and the purity was verified by resolving the protein using 12% SDS-PAGE, followed by staining with Bio-SafeCoomassie® G-250 (Simply Blue SafeStain, Thermo Fisher Scientific). The integrity of the protein was established by western blotting, using an anti-His6-HRP conjugated MAb.

### CPA toxin biological activity with EYDT assay

The phospholipase C (originally called lecithinase) activity of the recombinant CPA toxin and bacterial culture supernatants was assayed in an egg yolk agar well diffusion turbidity (EYDT) test [28]. A tryptose sulphite cycloserine (TSC) agar plate supplemented with 2% (vol/vol) egg yolk was punched out using the back of a 20–200 μl pipette tip, to obtain holes with a diameter of 7 mm. Twenty microliters containing from 0.5 to 5 μg of the deleted rBacCPA250–363H6 protein and tested undiluted supernatant was added to each hole, along with 4 mM of calcium chloride. Commercial full-length phospholipase C (PLC) toxin at 0.5, 1, 2, 5 μg was used as standard (P4039 Sigma). Plates were incubated at 37 °C for 24 h and the diameters of the zones of opacity were measured using a calibre. The lecithinase activity (U/ml) was quantified using a standard curve, in which the log of concentration of the PLC standard was plotted against the radial diffusion diameter of turbidity [29].

### Cell culture

Human colon carcinoma cells (CaCo-2) were purchased from ATCC® HTB-37™. Cells were cultured in DMEM (Thermo Fisher) and supplemented with 15% FBS, according to the instructions provided in the manual. All the incubation steps were carried out at 37 °C in the presence of 5% carbon dioxide [30].

### In vitro cytotoxicity assay

The toxicity of clostridial CPA toxin toward cultured eukaryotic cells has been previously reported [31, 32]. The activity of the deleted recombinant protein rBacCPA250–363-H6, a full-length PLC protein (Sigma 4039) and that of culture supernatant of the *C. perfringens* strain was evaluated in the CaCo-2 cell line. Briefly, 100 μl of a solution, comprised of 3 × 10 ^5^cells were seeded into each well of a 96 well plate and incubated at 37° for 24 to 36 h, until the cell monolayer exhibited almost 100% confluence. The medium was then removed from the wells and replaced with 100 μL of MEM without phenol that contained the different samples to be tested. Different concentrations of the truncated rBacCPA250–363-H6 toxin and the PLC protein (10 ng, 100 ng, 1 μg, 5 μg, 10 μg, 20 μg, and 50 μg) were tested. The sterile bacterial culture supernatant was diluted 2-fold from 1:2 to 1:8 (v/v) and added to wells in duplicate. The control wells were replaced only with MEM medium. Cytotoxicity was evaluated microscopically after 2 and 24 h. Protein toxicity was established by determining the metabolic activity of cells using the MTS assay. Briefly, tetrazolium [3- (4,5-dimetil-2-il) -5- (3-carbossimetossifenil) -2- (4-sulfofenil)-2H-tetrazolium](MTS) reagent was added to cells, according the manufacturer’s protocol (Promega) and the absorbance was measured at 490 nm after 2 h. Cell viability was compared against untreated and positive controls, in order to assess the percentage of metabolically active cells [33]. The protein dose that caused 50% of cell mortality (CT50) was defined as the toxin concentration that reduced the optical density (OD) recorded at 490 nm by 50%, against that of the untreated control.

### Generation of polyclonal and monoclonal antibodies

Rabbit polyclonal antibody sera (PAbs) and monoclonal antibodies (MAbs) were obtained by the immunisation of rabbits and Balb/c mice, respectively, with the purified truncated recombinant rBacCPA250–363-H6 protein. The antibodies were developed from external custum service the PRIMM Biotech Company (Dosson di Casier, Treviso, Italy), that declare to follow good laboratory practices during experimentation and the standard operating procedures of the facility and D. LGS 4 March 2014, No. 26 (Italian transposition of Directive 2010/63 EU on the protection of animals used for scientific purposes). Rabbit and mouse sera and hybridoma supernatants were screened by indirect ELISA to assess the production level of specific antibodies. The MAb isotype of antibodies was determined according to the manufacturer’s protocols (PRIMM Biotech SRL). The selected hybridoma cell lines were delivered to the lab and stored at − 80 °C [34]. Monoclonal antibodies were produced in hybridoma tissue culture supernatant, in IMDM (Iscoves medium w/glutamax-I–Thermo Fisher) medium supplemented with 1% FBS, according to the procedure outlined in laboratory manuals [35, 36]. MAbs were purified (rProtein A GraviTrap, GE Healthcare) and quantified using the Bradford method.

### Characterization of antibodies by Immunoblot assay

Samples containing truncated rBacCPA250–363H6 (0.1 μg), full-length PLC protein (0.1 μg), *C. perfringens,* and *C. septicum* culture supernatants were denatured and separated by a 12% SDS-PAGE, as previously reported. The resolved proteins were transferred to a PVDF membrane and blocked by incubating it with TBST containing 5% (w/v) non-fat milk for 2 h at room temperature (RT). The membrane was washed and incubated ON at 4 °C with the PAbs and each of the MAbs, that were derived from distinct hybridoma cell lines, after diluting them in TBST containing 3% skim milk. A commercial rabbit anti-phospholipase C polyclonal antibody (CRE-CABT-L1999- Star Fish) was also tested for comparing the results with those observed for the developed PAbs. After four washes, the membrane was incubated with secondary HRP conjugated, anti-mouse, or anti-rabbit antibodies, according to the nature of the sample tested. The antibody-antigen interactions were highlighted with the SuperSignal West Pico Chemiluminescence kit (Thermo Scientific).

### In vitro neutralization of CPA toxin biological activity

The ability of the antisera and developed MAbs to neutralize the phospholipase C activity of full-length PLC toxin was assessed in EYDT assay. The negative control was obtained from mouse sera before carrying out immunization. Serially diluted serum (from 1:2 to 1:960) and an increasing concentration of MAb (from 5 to 120 μg) were pre-incubated at 37 °C for 1 h with a constant amount of PLC toxin (1 μg) and 4 mM of calcium chloride, and PLC activity was then measured in egg yolk lipoproteins, as described previously. After incubation, the variations in radial diffusion diameter were measured and compared to those of the control.

### In vitro neutralization cytotoxicity assay

The ability of a specific monoclonal antibody (MAb 318F11B7) and antisera raised against rBacCPA250–363H6 to neutralize the cytotoxicity of full-length PLC toxin and *C. perfringens* culture supernatant (C-5560/18) was established by an in vitro assay. Briefly, the sera and MAb, were diluted at different concentrations and pre-incubated for 1 h at 37 °C, with the PLC toxin present at a concentration corresponding to its CT50 (50 μg /ml) value Then the solution was applied, in triplicate, to wells with a confluent cell monolayer, to assess each condition that needed to be examined. After 16–24 h, the CaCo-2cell viability was measured by the MTS assay.

## Results

### Production and purification of the truncated rBacCPAH6 proteins

**Fig. 1:**
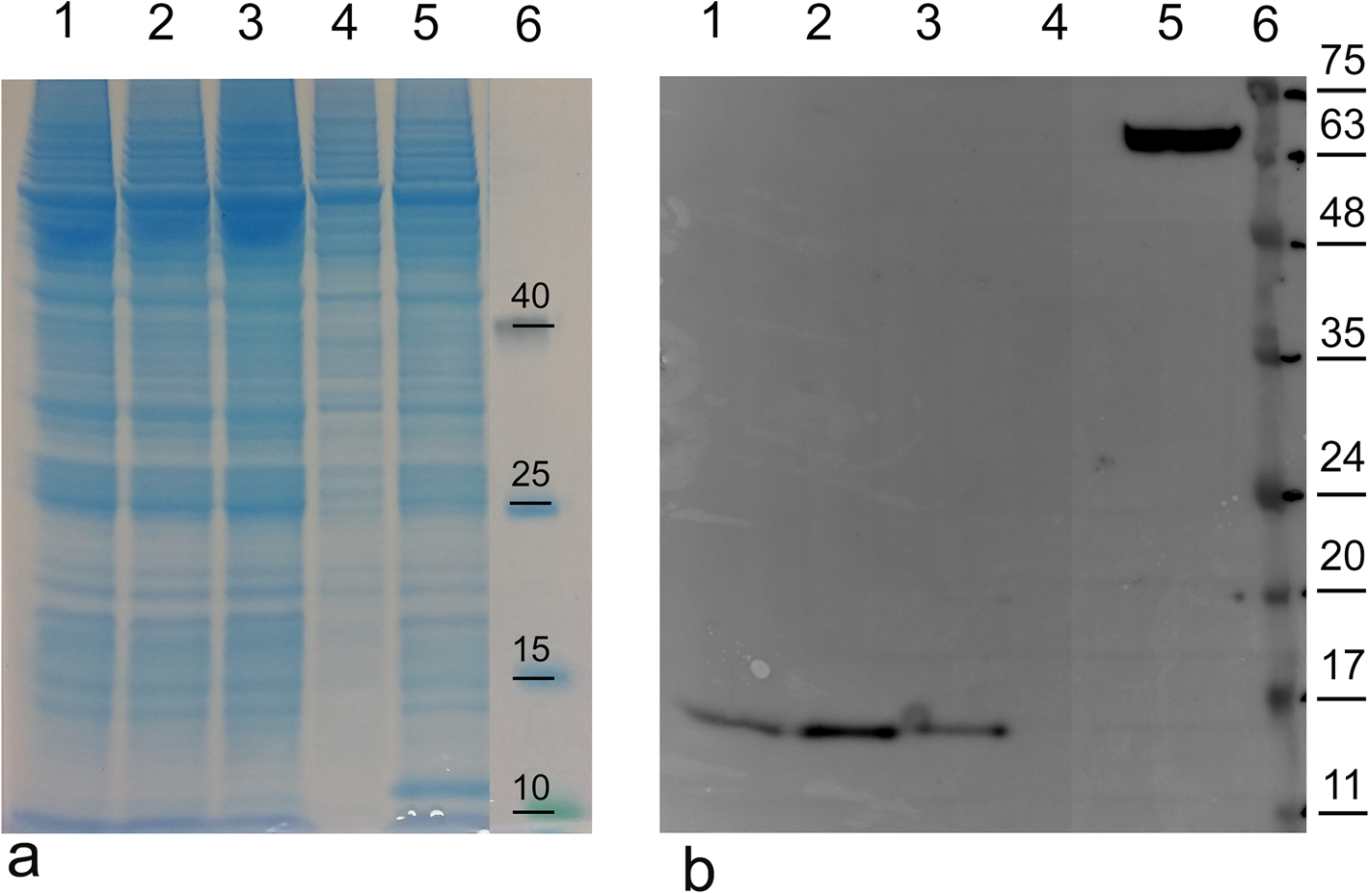
Analysis of the C-terminal fragment rBacCPA279–363H6 with different consensus leader sequences. 7.5 × 10^6^ Sf21 cells were infected in 10 ml of an EX-Cell 420 serum free medium with recombinant virus at a multiplicity of infection (MOI) of 2.0. After 72 h post-infection the infected cells were collected and lysed and the total protein of this preparation was quantified by the Bradford assay. Equal quantity of the cellular lysate of each sample was loaded electrophoresed on 12% SDS-PAGE gels and at the same time both (**a**) stained with Coomassie blue and (**b**) analysed in Western blot using an anti-His6-HRP conjugated MAb. Lane 1: rBac CPA279–363H6 without a leader sequence; lane 2: rBacCPA279–363H6 with *L21* sequence; lane 3: rBacCPA279–363H6 with *Kozak*s sequence; lane 4: non-infected Sf21 cells (negative control); lane 5: Sf21 cell infected with recombinant baculovirus containing a target gene His tagged but not related with CPA (His6 positive control); lane 6: protein markers (the molecular weight bands in kDa, are reported on the right). The infections was repeated more than three times and the figure represents the highest-quality picture obtained

To establish the effect of the different consensus leader sequences on the protein expression grade and optimal growth conditions, an initial investigation was performed with rBacCPA279–363H6 protein, which was the first truncated recombinant toxin to be generated (Fig. 1).

**Fig. 2:**
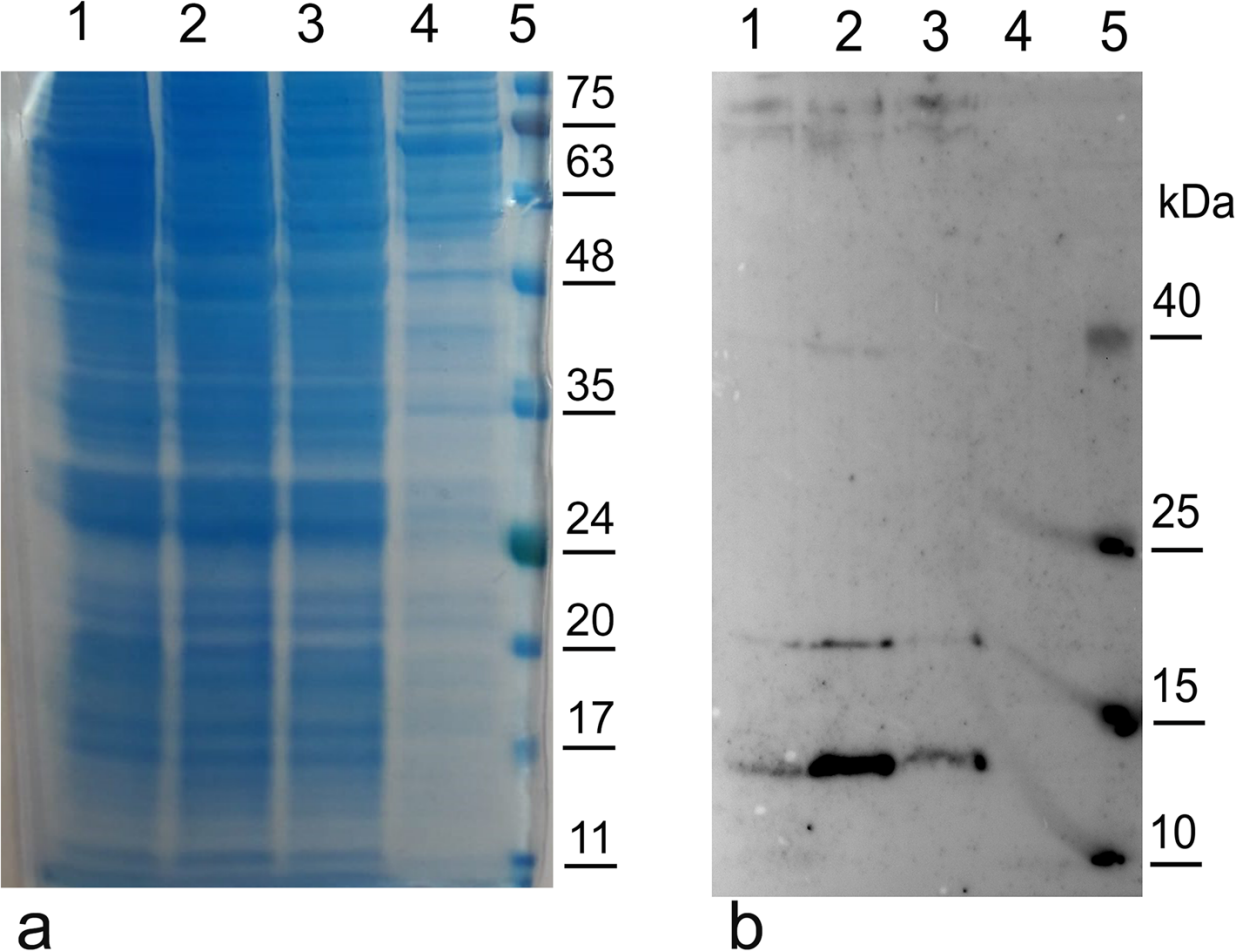
Western blot analysis of the C-terminal fragment rBacCPA279–363H6 in different media. Sf21 cells were infected with rBacCPA279–363H6 with *L21* sequence in different media and the cellular lysates harvested 72 h post-infection and quantified by the BioRad assay. Equal quantity of the cellular lysate of each sample was loaded electrophoresed on 12% SDS-PAGE gels and simultaneously both (**a**) stained with Coomassie blue and analysed (**b**) in Western blot using an anti-His6-HRP conjugated MAb. Lane 1: recombinant protein in Grace’s medium supplemented with 10% FBS; lane 2: recombinant protein in EX-cell 420 serum free medium; lane 3: recombinant protein in HYQ-SFX in serum free medium, lane 4: cellular lysate of Sf21 uninfected cells (negative control); lane 5: protein markers with the molecular weight bands in kDa reported on the right

Three recombinant plasmids, i.e., pOET2-CPA279–363H6, pOET2-*Kozak*CPA279–363H6, and pOET2-*L21*CPA279–363H6, which differed with regard to the presence/absence of *Kozak* and *L21* leader sequences upstream of the start codon, were constructed. Using general DNA cloning protocols and a PCR strategy, the N-terminal domain, which included the putative signal sequence (first 28 aa) of the *cpa* toxin gene, was removed and part of the domain (from 279 to 363 aa) was cloned *in frame* with 6xHis tag into the pOET2_C-6XHis vector. The analysis of the nucleotide sequences, performed for all the constructs obtained with these cloning procedures, showed a perfect identity in the nucleotide composition (data not shown). The proteins were expressed in Sf21 insect cells and analysed by western blotting, usinganti-His6-HRP conjugated MAbs. The MAbs revealed the presence of immunoreactive bands with a molecular weight of approximately 11 kDa in the cellular extract, as expected after the elimination of the signal sequence (Fig. 1). Moreover, a different expression level has been shown according to the regulatory sequences; *Kozak* and especially the *L21* sequence induced an enhancement in protein expression levels. The comparison of the level of rBacCPA279–363H6 produced in three different growth mediums, i.e., Grace’s medium supplemented with 10% FBS, HyClone-SFX, and Ex-cell 420 has highlighted the fact that the use of the Ex-cell 420 culture medium resulted in the best performance and guaranteed that the protein production process would be more efficient (Fig. 2).

**Fig. 3:**
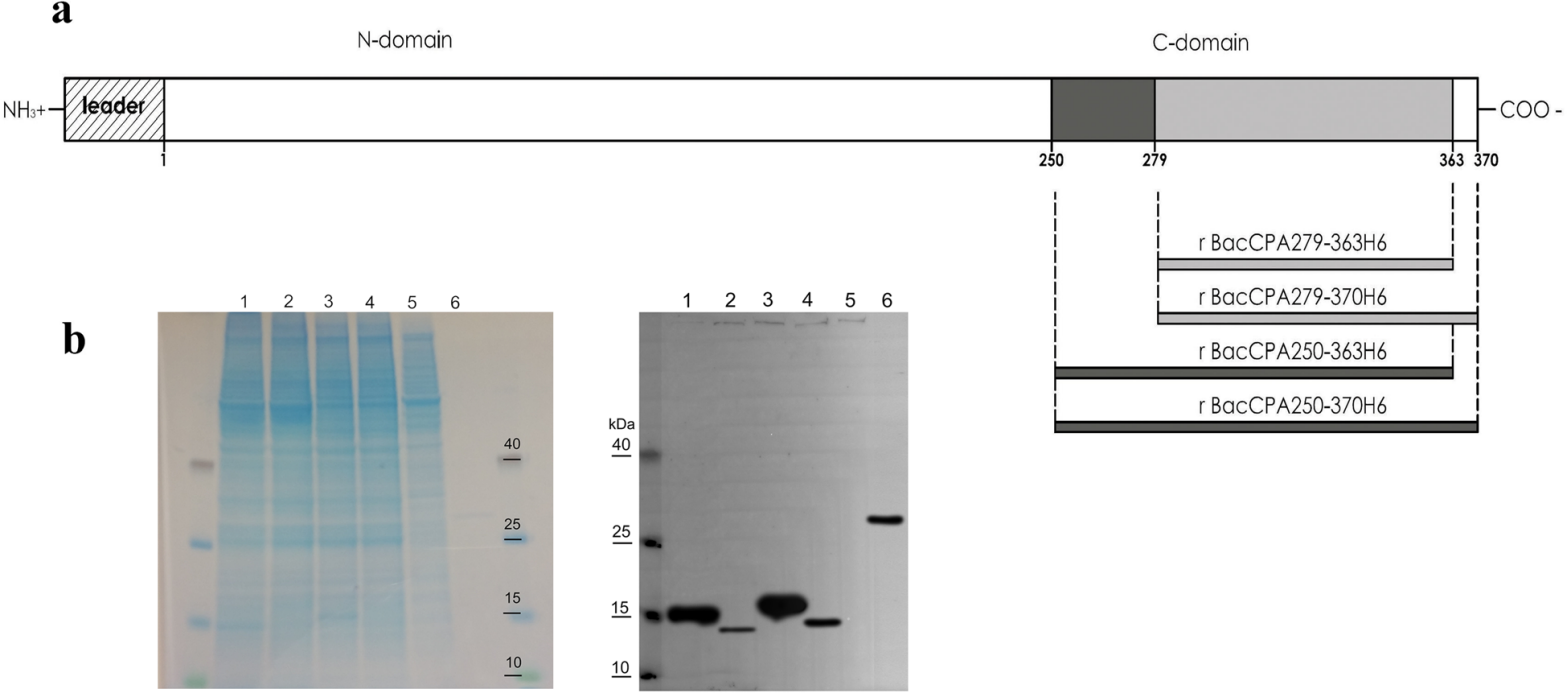
Schematic representations and analysis via Western blot of the truncated recombinant rBacCPAH6 toxins. **a** The scheme were drawn approximately on scale, with amino acids numbered according to their positions in the CPA. Leader peptide (amino acids 1–28) were depicted as shown. The deleted forms of rBacCPAH6 were represented by bars. **b** Equal quantity of the cellular lysate of each sample harvested 72 h post infection, was loaded, electrophoresed on 12% SDS-PAGE gels and stained with Coomassie blue and analysed in Western blot using an anti-His6-HRP conjugated MAb. Lane 1: rBacCPA250–363H6 (approximately 15 kDa); lane 2: rBacCPA279–363H6 (approximately 11 kDa); lane 3: rBacCPA250–370H6 (approximately 16 kDa); lane 4: rBacCPA279–363H6 (approximately 12 kDa); lane 5: cellular lysate of Sf21 uninfected cells (negative control); lane 6: 100 ng of purified rCPB2Δ1–25-His6 (His6 positive control). The protein molecular weight marker is reported

**Fig. 4:**
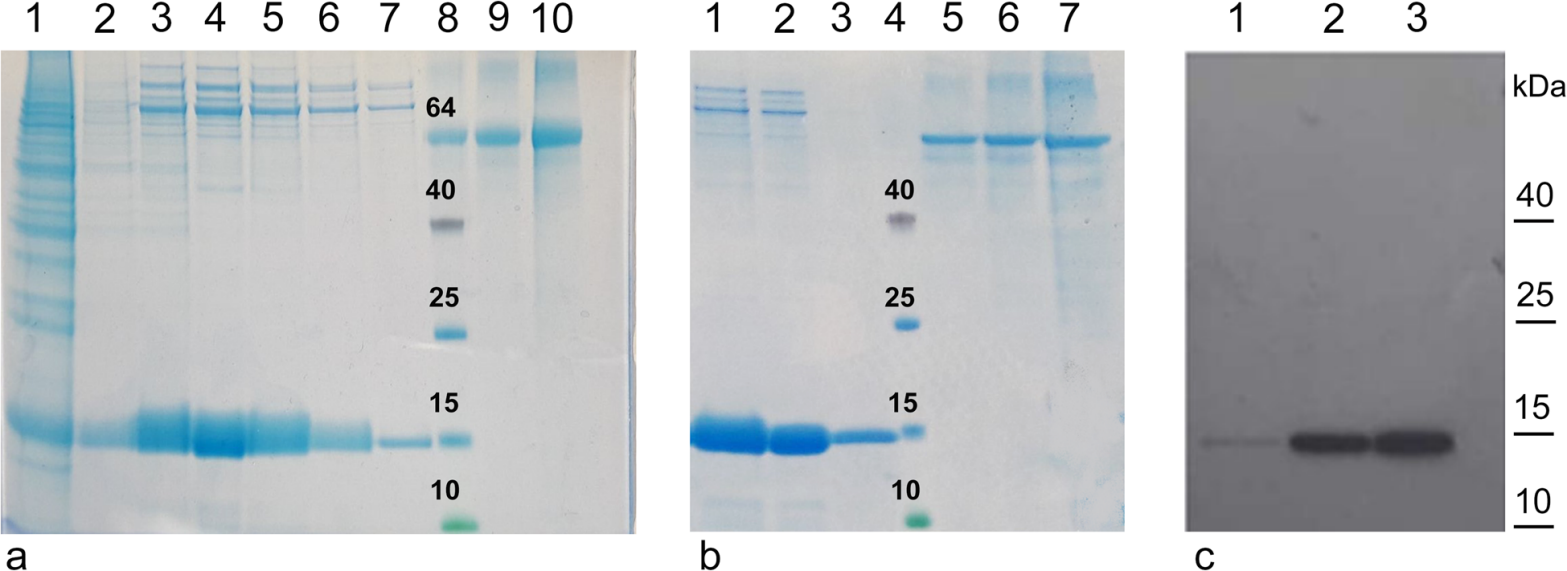
Purification and characterization of truncated recombinant rBacCPA250–363H6. **a** Coomassie stained 12% SDS-PAGE analysis after Ni-NTA purification. Lane 1: fraction observed on washing the column, after the passage of the recombinant rBacCPA250–363H6; lane 2–7: Elution fractions; lane 8–10: 1 μg, 2 μg and 4 μg of BSA standard; lane 8: Protein molecular weight marker. **b** Coomassie stained 12% SDS-PAGE analysis after Ni-NTA purification and after the dialysis step in PBS buffer. Lane 1: Elution fractions before the dialysis step (10 μl); Lane 2: Elution fractions after the dialysis step (10 μl); Lane 3: Elution fractions after the dialysis step (1 μl); (**c**) Immunoblot analysis of the elution fractions performed after Ni-NTA purification using anti-His6-HRP conjugated MAb. Lane1–3: Elution fractions corresponding to lane 2, 3 and 4 of samples in Comassie (**a**). The protein molecular weight marker is shown on the right

The recombinant rBacCPA279–363H6 protein was generated on a large scale in EX-cell 420 medium, using a baculovirus with the *L21* signal sequence and it was purified using the Ni-NTA system under different experimental conditions. Several attempts for protein purification were made, to obtain the protein in native and denaturing conditions; however, a satisfactory yield and level of purity could not be achieved (data not shown). Hence, three new truncated recombinant proteins, longer than the first (from 250 to 370 aa), were designed, i.e., CPA279–370, CPA250–363 and CPA250–370 and were expressed in insect cells. All the baculoviruses generated contained the *L21* signal sequence upstream of the start codon and the corresponding proteins were produced in the serum free Ex-Cell420 medium in equivalent infection conditions (Fig. 3). Western blotting analysis with anti-His6-HRP conjugated MAb revealed that the molecular weights of immunoreactive bands representing the presence of rBacCPA279–363H6, rBacCPA279–370H6, rBacCPA250–363H6, and rBacCPA250–370H6 were approximately 11, 12, 15, and 16 KDa, respectively. Under the same infection conditions, the first and second recombinant proteins were expressed at very low levels (Fig. 3b, lane 2–4), as compared to the expression levels of the two other recombinant toxins (Fig. 3b, lane 1–3). Therefore, based on these results, the baculovirus containing the *L21* sequence that expressed the truncated recombinant protein rBacCPA250–363H6 was selected for use in subsequent studies. Ni-NTA purification was performed in both native and denaturing reducing conditions; however, a satisfactory yield, purity, and protein concentration was achieved only with the latter. Figure 4a shows the process of purification of recombinant rBacCPa250–363H6 and Fig. 4b the eluition fractions before and after dialisys step, using Coomassie stained SDS-PAGE, and the specificity and integrity of the protein, which were determined using immunoblot analysis with anti-His6-HRP conjugated MAb (Fig. 4b). In conclusion, after the dialisys step a degree of purity that was ≥85% was obtained with a yield of 4 mg for 10^9^ insect cells.

### In vitro cytotoxicity

**Fig. 5:**
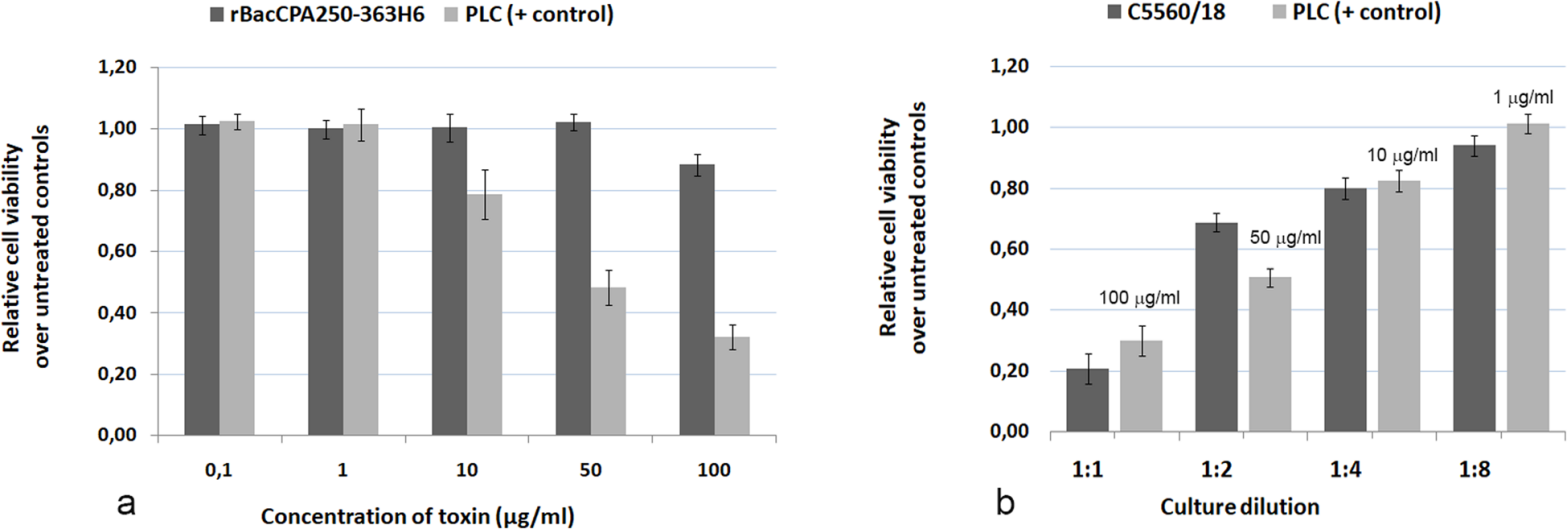
Cytotoxicity of rBacCPA250–363H6 in CaCo-2 cells. Cells were treated as described in the methods section with rBacCPa250–363H6, PLC and culture supernatant of *C. perfringens* C-5560/18 strain. **a** The viability of cells, as determined by the MTS assay, after treating cells with indicated concentrations of rBacCPA250–363H6 and PLC toxins and (**b**) with a serially diluted solution of the supernatant of *C. perfringens* C-5560/18 strain. The data represent the mean of three independent experiments

The cytotoxic activity of the rBac250–363CPAHis6 protein was tested in parallel with that of the full length PLC protein and *C. perfringens* culture in CaCo-2 cells using the MTS assay. No morphological changes were observed, as compared with the untreated control cells, after cells were exposed to doses of 0.1, 1, 10, 50, and 100 μg/ml of rBac250–363CPAH6 (Fig. 5a). However, morphological changes, including the rounding up of cells, were observed in cells exposed to 10, 50, and 100 μg/ml of PLC toxin (data not shown).

The MTS assay results revealed that the viability score of CaCo-2 cells treated with up to 50 μg/ml rBacCPA250–363H6 was 100%, while that of cells treated with 100 μg/ml of recombinant protein was 88% (Fig. 5a). Notably, the presence of the PLC protein lowered cell viability to 80%, when a dose of 10 μg/ml was used, and to 33%, when a dose of 100 μg/ml was used (Fig. 5a). Interestingly, the undiluted culture supernatant of *C. perfringens* C5560/18 induced similar cytotoxic effects as those observed using 100 μg/ml of PLC and led to a drastic reduction in cellular metabolic activity, as compared to that in untreated control cells (Fig. 5b). No cytotoxic activity was observed for the truncated rBacCPA250–363H6 protein at concentrations till to 50 μg/ml; instead, for the full-length PLC, cytotoxicity was observed at a concentration that was as low as 10 μg/ml, and the CT50 was determined at 50 μg /ml.

### In vitro neutralization of CPA toxin biological activity

**Fig. 6:**
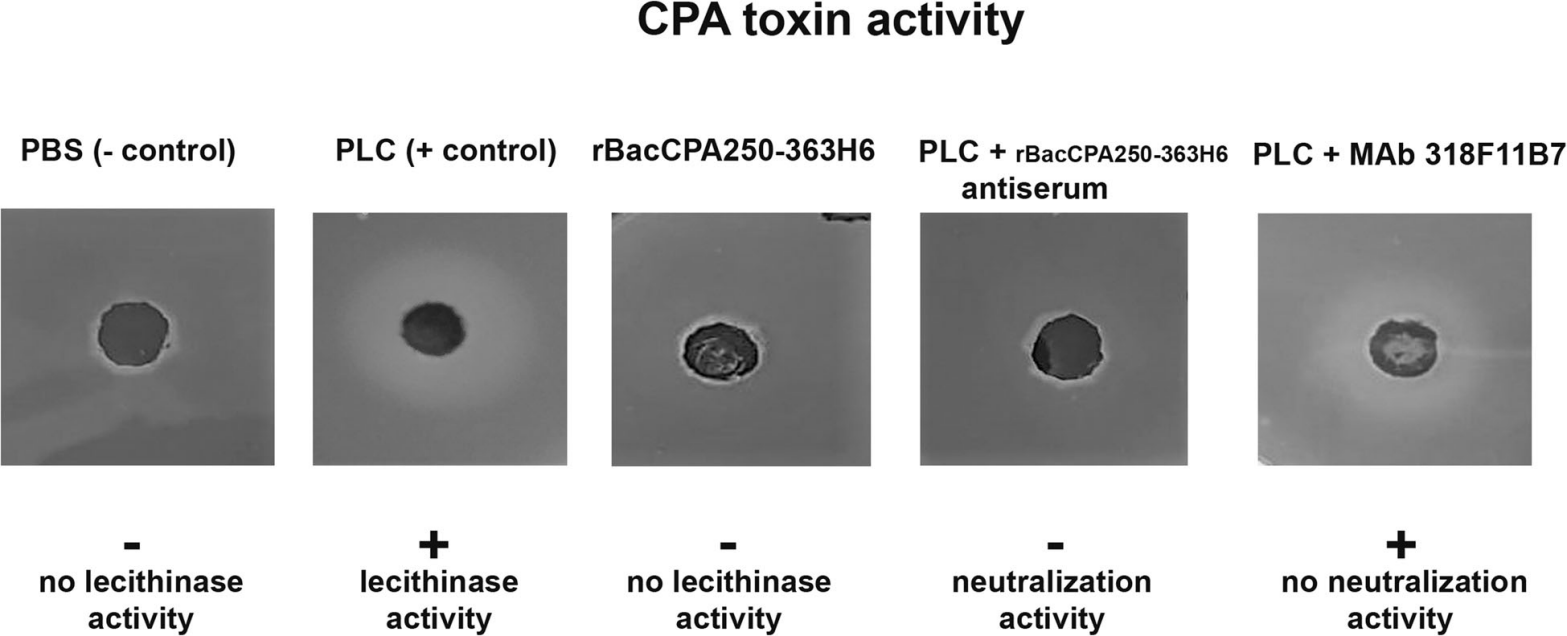
In vitro neutralization of the biological activity CPA toxin. Full-length PLC were left untreated or pre-incubated, in the presence of 4 mM CaCl_2_, with serum obtained from a mouse immunized with the non-toxic rBacCPA250–363H6 and the specifically generated MAb 318F11B7. They were then spotted on TSC agar and incubated overnight at 37 °C. The neutralization of PLC activity results in the absence of an outer zone of hydrolysed phospho-lipid. The positive symbol (+) indicate the presence the activity of the toxin while the negative symbol (−) indicate the absence of the activity of the toxin or its complete neutralization. Recombinant truncated rBacCPA250–363H6 protein untreated did not exhibit any lecithinase activity. Representative image of three independent experiments is shown

The capability to break down the lecithin present in the egg yolk and the resulting white halo around the colonies was used as a measure of CPA biological activity. The truncated recombinant rBacCPA250–363H6 protein did not exhibit any lecithinase activity at all the tested concentrations (Fig. 6). However, on using different concentrations of full-length PLC as standards (from 0.5 to 5 μg), the radial diffusion diameter varied between 13 to 23 mm. The culture supernatants of different *C. perfringens* strains were tested and only in three strains the CPA toxin activity was detected as 30 10^− 3^ U/ml. To determine whether the MAb 318F11B7 and the immune serum obtained against the rBacCPA250–363H6 proteins could neutralize the phospholipase activity of full-length PLC toxin, serially diluted antisera and increasing concentrations of MAbs were incubated with 1 μg of PLC and tested using the EYDT assay. When the antisera of rBacCPA250–363H6 were used at a final dilution of up to 1:80, the PLC activity was completely neutralised, instead, MAb 318F11B7 was unable to neutralise the activity of PLC at all the tested concentrations (Fig. 6).

### Characterization of PAbs and MAbs

**Fig. 7:**
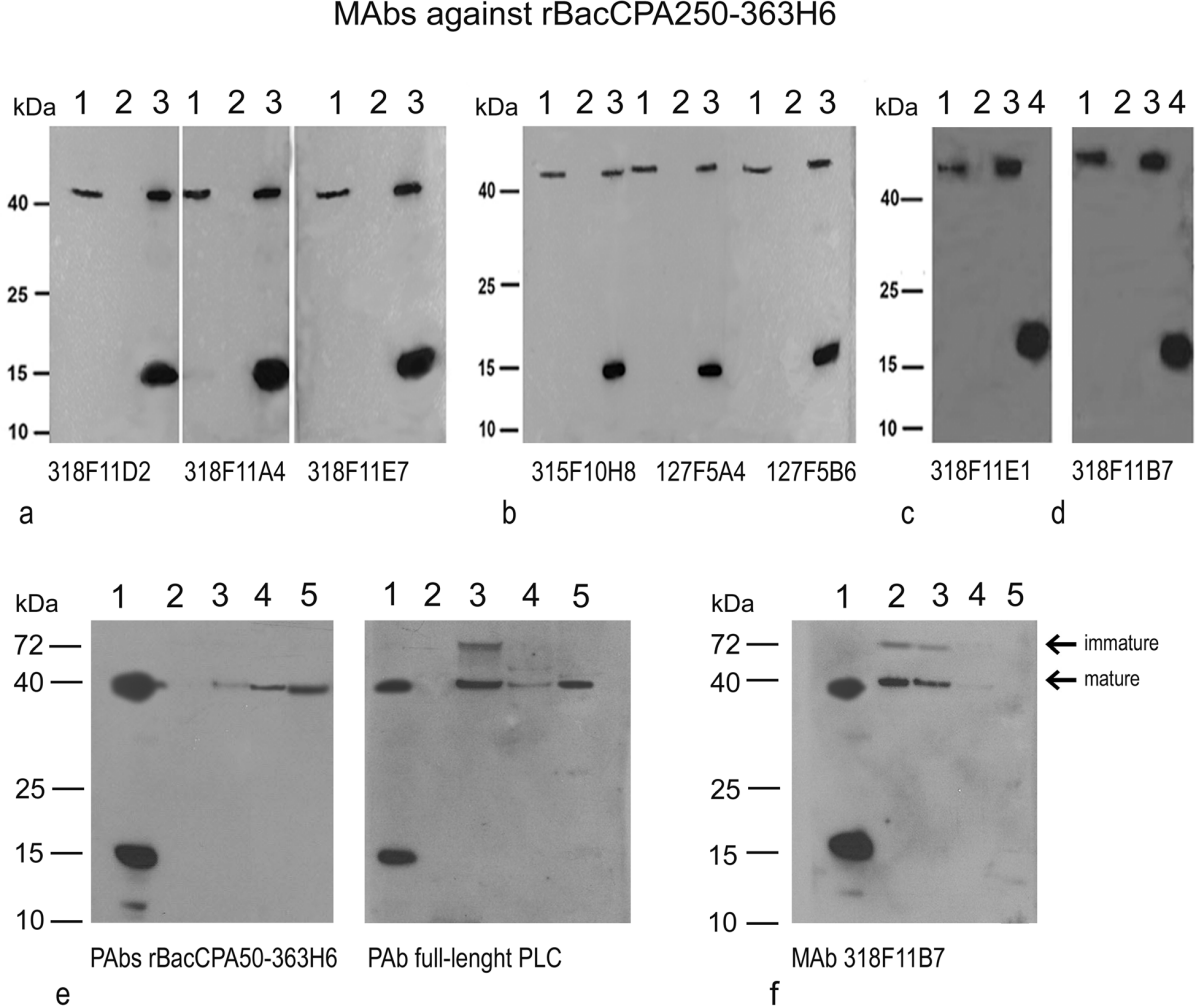
Characterization of polyclonal and monoclonal antibodies against rBacCPA250–363H6 by western blotting. The MAbs (section **a**-**d**) and PAbs (section **e**) generated were tested against culture supernatant of the *C. perfringens* CPA toxin producer. The name of the tested antibody is shown below the corresponding picture. The MW marker is shown on the left. Section **a** and **b**: Culture supernatant of *C. pefringens* strain C-5560/18 (lane 1), culture supernatant of *C. septicum*, used as a negative control (lane 2), and a mixture of full length PLC and rBacCPA250–363H6 (lane 3). Section **c** and **d**: Culture supernatant of *C. perfringens* strain C-5560/18 (lane 1), culture supernatant of *C. septicum*, used as a negative control (lane 2), full length PLC (lane 3), and rBacCPA250–363H6 (lane 4). Section **e**: Rabbit anti-rBacCPA250–363H6 PAbs and anti-PLC (Star Fish): mixture of rBacCPA250–363H6 and PLC (lane 1), different culture supernatants of *C. perfringens* CPA protein producers (lane 3–5), and culture supernatant of *C. septicum*, used as a negative control (lane 2). Section **f**: MAb 318F11B7 tested against recombinant and native CPA: mixture of rBacCPA250–363H6 and PLC (lane 1), different culture supernatants of *C. perfringens* CPA protein producers (lane 2–4), and culture supernatant of *C. septicum*, used as a negative control (lane 5)

Two polyclonal and eight monoclonal antibodies were obtained against the truncated recombinant protein rBacCPA250–363CPAH6. The immunogenicity of the protein was evidenced by the seroconversion of animals and evaluated by an indirect ELISA test; additionally, the reactivity of PAbs and MAbs was evaluated using an ELISA test (data not shown) and immunoblot assay (Fig. 7), conducted using the truncated rBacCPa250–363H6, full-length PLC, and *C. perfringens* culture supernatants as antigens.

The resultant MAbs and PAbs recognized an immunogenic band with a molecular weight of 15 KDa from the lane containing truncated rBacCPA250–363H6 and a band with a size of 43 KDa corresponding to the full-length PLC and native CPA toxin in culture supernatants. The antibody specificity was tested using a *C. septicum* culture. Different levels of reactivity were observed among the eight monoclonal antibodies obtained: the MAbs315F10H8, 127F5A4 and 127F5B6 showed moderate reactivity, while the remaining five MAbs, i.e., 318F11D2, 318F11E7, 318F11A4, 318F11B7, and 318F11E1 showed strong reactivity in the western blot towards r250–363CPA His6 and native PLC (Fig. 7 a-d). Fresh culture supernatants of different *C. perfringens* strains were tested using the MAb 318F11B7 (Fig. 7f), and in addition to the expected band at 43 KDa, another high molecular weight band (about 70–80 KDa) was observed, whose presence was probably attributable to a precursor or dimeric forms of native CPA. In Fig. 7e, anti-rBacCPA250–363H6 PAbs and a polyclonal commercial Rabbit Anti-Phospholipase C were tested to compare them. All the polyclonal antibodies highlighted the presence of the recombinant protein r250–363CPAHis6 and wild-type CPA protein at the same height as the PLC. MAb 318F11B7 exhibited the ability to neutralize the cytotoxicity of full-length PLC in CaCo-2 cells. For the in vitro neutralization assay, 1x CT50 of PLC toxin (50 μg/ml) was pre-incubated with diluted sera and different concentrations of MAbs at 37 °C for 1 h, and the mixture was then applied to the cells. The MTS assay results demonstrated that cells were only partially protected from the cytotoxicity of PLC. In the experimental conditions adopted, the use of merely100 μg/ml of MAb resulted in PLC toxicity of up to 14%, as compared to that in untreated control cells (data not shown).

## Discussion

Clostridial myonecrosis, also known as gas gangrene, is a devastating histotoxic infection caused in humans and animals by *C. perfringens* strains. The high incidence, extensive morbidity, and high lethality rate, associated with Clostridiosis infections represents a serious concern for livestock, as it is responsible for significant economic losses that are particularly linked to a high level of neonatal mortality. Due to the rapid course of the disease, curative treatment is not possible; therefore, vaccination is the best prophylactic measure for this disease. The virulence of *C. perfringens* is attributable to the presence of its several extracellular toxins. The CPA toxin is produced by all types of *C. perfringens* strains and is an important immunogenic antigen that is involved in the pathogenesis of enterotoxaemia, and in the induction of necrotic lesions in the calf intestinal loop model [19]. The role of the type A strain in enterotoxaemia is rather controversial and has been extensively debated. Recently, it was shown, that neutralizing antibodies raised against the native CPA form and formaldehyde inactivated toxoid provided protection against *C. perfringens* infections [19, 37]. Although this was an efficient strategy, the use of the active toxin was not safe and formaldehyde inactivation was required, even though it reduced immunogenicity [38, 39], and a risk of residual toxicity and hypersensitivity was associated with the presence of residual formaldehyde. The production of traditional clostridial vaccines involves the detoxification, purification, and antigen concentration steps; the potency of these vaccines varied from batch to batch and biosafety measures were required to manage the *C. perfringens* culture. In recent years, owing to the development of new genetic techniques, and the increased adoption of tridimensional studies and structural vaccinology, researchers have focused on developing more methods of producing recombinant proteins for use in veterinary vaccines. Studies of tridimensional structures and the functional characterization of the CPA toxin revealed the presence of two separate domains, a catalytic N-terminal domain, and a smaller membrane-binding C-terminal domain. Only the latter is immune-protective, in fact, Nagahama et al. demonstrated that mice immunized with GST-CP251–370 and GST-CP281–370 provided protection against the lethal effects of alpha toxin [18] and also ensured the survival of animals injected with 10 LD_100_ doses of viable *C. perfringens* type A [17]. Recently, Shreya et al. reported that the immunization of mice with a bivalent chimerical protein Cpae (composed of the C-domains of CPA-toxin and enterotoxin CPE) resulted in a substantial level of protection against *C. perfringens* type A toxaemia [40]. Therefore, a C-terminal domain lacking in any enzymatic and toxin activity might be the principal immunogen of an alpha-toxin vaccine [19]. The reasons why the C-terminal domain acts as a protective immunogen are not completely clear, but it is possible that an antibody against this domain had blocked the initial membrane-binding event [37].

In this study, several truncated forms of the CPA toxin (truncated rBacCPAH6) were generated for the first time in a heterologous expression system, such as Sf21 insect cells, using baculoviruses. The baculoviruses were able to produce the truncated rBacCPA279–363H6, rBacCPA279–370H6, rBacCPA250–363H6, and rBacCPA250–370H6 proteins, all lacking the N-terminal domain and the 28 amino acids (aa) of the putative signal sequence. The choice to use these new attenuated recombinant viruses (produced using the *flash*BAC system) guarantees robustness, cost-effectiveness, high levels of expression, and a higher degree of biosafety.

Previous studies have shown that the expression levels of exogenous genes in baculovirus-infected insect cells were increased significantly in the presence of specific leader sequences [23, 41]. Hence, an initial investigation was performed to determine the level of expression of the rBacCPA279–363H6 protein that infected S21 cells, using three baculoviruses that differed with regard to the absence and presence of the *Kozak* sequence and the presence of *L21* leader sequences upstream of the start codon. Figure 1 shows that the *Kozak* sequence and especially *L21* leader sequences are essential for enhancing the expression of recombinant proteins. Moreover, studies performed to determine the optimal medium and infection conditions demonstrated that Ex-cell420 was the most suitable growth medium for maintaining viable the infected cells for at least three days (Fig. 2). The baculovirus expression system is often chosen for its ability to enable post-translational modifications to be made, such as those involving N-glycosylation [42]; hence, an analysis of the amino acid sequence of the C-terminal domain of the CPA toxin was performed by the NetNGlyc 1.0 server. The results of this analysis predicted the presence of a hypothetical N glycosylation site at Asn-Xaa-Ser/Thr, between the aa residues 364 to 370; thus, in order to avoid undesired glycosylation, this region was also removed in two constructs (rBacCPA279–363H6 and rBacCPA250–363H6). However, none of the four truncated recombinant rBacCPAH6 proteins (all generated from baculoviruses containing the *L21* signal) were glycosylated, in fact, no increase in electrophoretic mobility was observed and the western blot analysis revealed the presence of immune-reactive bands with expected molecular weights of 11, 12, 15, and 16 KDa (Fig. 3). Under the same infection conditions, expression of two proteins with the amino acid residue 279 (rBacCPA279–363H6 andrBacCPA279–370H6) at the start of the sequence was observed to be very low, as compared to that of proteins containing the aa residue 250 (rBacCPA250–363H6 and rBacCPA250–370H6) at the start of the protein sequence. The recombinant rBacCPAH6 proteins lacked all the N-terminal regions and the signal sequence, which represented by the first 28 amino acids, and were intracellular proteins occurring in the cellular lysates; in contrast, native CPA was present in the culture supernatant of *C. perfringens*. In addition, the best results were achieved in terms of production, satisfactory yield, and purity of the rBacCPA250–363H6 protein, when the cellular lysate was subjected to Ni-NTA His-select–affinity chromatography under denaturing conditions. As suggested for other recombinant proteins, such as CPB2 [24], this feature could be explained with respect to the folded protein by the significant exposure of the C-terminal 6xHis tag of the denatured antigen. As shown in Fig. 4, Coomassie stained SDS-PAGE and immunoblotting analysis confirmed the high degree of purity (more than 85%), specificity, and integrity of the protein.

Previous studies have established that the alpha toxin has cytotoxic effects on bovine endothelial cells, acting in synergistic effect with PFO, in fact, almost all cells detached after exposure to supernatant of the wild-type gas gangrene *C. perfringens* strain [43]. Endothelial cells form a vital barrier that controls the exchange of cells, macromolecules and fluids between the vascular lumen and the surrounding tissue. Disruption of the endothelial barrier leads to increased vascular permeability along with tissue edema and hemorrhage. CPA stimulate leucocyte adherence, probably by increasing vascular leucostasis and local ischemia [6]. The human enterocyte-like CaCo-2 cell line has been used extensively over the last twenty years as a model of the absorptive and defensive properties of the intestinal mucosa [44, 45]. Shreya et al. has demonstrated that recombinant Cpae chimera it bound to CaCo-2 cell membrane but did not shown any toxicity [40]. We evaluated the cell viability of CaCo-2 cells treated with the truncated recombinant CPA and full-length PLC proteins, and compared them with that of untreated and positive controls, in order to assess the percentage of metabolically active cells. Cytotoxicity studies were performed on intestinal epithelial cell lines using rCPA250–363H6, and they confirmed that this antigen, which was produced in baculovirus systems, was non-toxic, in comparison to the full-length PLC and native toxin present in *C. perfringens* supernatant. In fact, no changes were observed in the morphological traits of CaCo-2 cells and a minimal cytotoxicity (about 10%) was reported after the MTS assay was performed using truncated recombinant CPA proteins present at a concentration of up to 100 μ/ml. Instead, CaCo-2 cell damage was observed after the confluent monolayer of cultured cells came into contact with 10 μg/ml of the full-length PLC or a culture supernatant that was diluted up to four times. The data were in accordance with the results obtained for the CPB2 atypical recombinant protein, which was expressed in baculoviruses [24].

The non-toxic recombinant rBacCPA250–363H6 protein purified under denaturing conditions was used to generate polyclonal and monoclonal antibodies (Fig. 7). Eight MAbs and two PAbs were obtained, and all of them recognized the truncated recombinant CPA (15 KDa), full-length PLC, and native CPA toxin secreted in culture supernatants (43 KDa), in the Western blot (Fig. 7a-e). MAbs showed high specificity and were unreactive against *C. septicum* culture supernatants. As showed in Fig. 7f, in addition to the predominant, mature form of CPA, the MAb 318F11B7 recognized a reactive band at a higher molecular weight (around 70–80 KDa), which could be attributed to dimeric forms of native CPA or precursor proteins with a signal sequence before post-translational processing that had been secreted in the *C. perfringens* supernatant, as suggested by Leslie et al. [46].

The neutralizing ability of the generated MAbs and mouse antisera was also tested through in vitro assays. Preliminary results showed that MAb 318F11B7 exhibited a partial ability to neutralize the cytotoxicity of full-length PLC in CaCo-2 cells. Under the experimental conditions used, the MAb resulted in a restoration of 14% cell viability only when a high concentration (100 μg/ml) of the MAb was used, as compared to that of the cells exhibiting toxicity with PLC and the untreated control (data not shown). This is in accordance with the findings of the study performed by Goossens et al., which found that *C. perfringens* induced endothelial cytotoxicity observed in vitro was very weakly neutralized by C-terminal fragment antisera [19]. Other researchers suggested that protection against the full length CPA toxin in vivo required only low molar ratios of neutralizing antibodies, whereas greater antibody to toxin molar ratios might be required to neutralise the cytotoxicity of PLC in vitro [32].

Alpha-toxin is a phospholipase C (lecithinase) that is capable of binding to the mammalian cell membrane in vivo, and cleaving membrane-bound phosphatidylcholine (or sphingomyelin) to produce phosphocholine and diacylglycerol (or ceramide). According to Moreau et al. [47], to maximize its activity, zinc is required for substrate hydrolysis and the calcium ion is essential for the binding of CPA toxin to the phospholipid monolayer, at concentrations of approximately 5 μM and 5 mM, respectively [12, 48]. The biological activity of CPA could be determined by an in vitro EYDT assay based on Nagler’s reaction. The non-toxic rBacCPA250–363H6 protein did not exhibit any lecithinase activity on TSC agar at all the concentrations tested, as compared to that of the native, full length PLC, which was used as a standard. Nevertheless, the antiserum against the recombinant C-terminal protein showed the ability to completely neutralize the PLC toxin activity in vitro (Fig. 6). The same inhibition was not observed with the use of MAb 318F11B7. In a recent study carried out in bovine enterotoxemia strains, was shown an activity of alpha toxin below the detection limit of 15.6 10^− 3^ U/ml and that were not significantly different from levels produced by strains isolated from healthy calves [29]. We detected an increased CPA activity only in the culture supernatants of three *C. perfringens* strains originating from enterotoxoemia cases and this in accordance with hypothesis that, a hither to unknown, *C. perfringens* virulence factor might be involved in the pathogenesis of calf enterotoxemia.

The polyclonal and monoclonal antibodies generated against rBacCPA250–363H6 were also characterized in an immune-enzymatic assay. In indirect ELISA, these antibodies recognized the recombinant rBacCPAH6 and full length PLC standard proteins, but showed a very low reactivity towards the native CPA observed in *C. perfringens* culture supernatants. These preliminary results, in combination with the good reactivity observed in the Western blot, suggested that MAbs target direct to linear epitopes, probably hidden in the native form of the toxin. This could be attributed to the purification of the antigen in denaturing conditions. In the reduced form, the protein might lack conformation-dependent epitopes, and steps to achieve refolding do not guarantee that the epitopes can be recovered on the protein structure. Some authors attributed the reduced reactivity of the antisera raised against the C-terminal domain to the presence of the tag, which was generally inserted in the recombinant protein to make the purification process easier [19]. This hypothesis could be proven for the GST tag, after the eventual distortion of the conformation of the toxin, but it is difficult to apply it to the His tag, because it is smaller than GST, and is less likely to influence the conformation of CPA. In accordance with other studies, where recombinant C-terminal domains of alpha toxin were generated in prokaryotic cells, we reported a N-terminal truncated rBacCPA250–363H6 protein produced in eukaryotic system that is enzymatically inactive; and though its lecithinase activity is drastically reduced and its toxicity is abolished, it maintains its antigenic proprieties [48]. Because of these characteristics, it is a promising candidate for the development of a new recombinant protein vaccine. After combining data from different studies conducted in different animal species with regard to the role of CPA toxin in enteric diseases, CPA emerged as an essential toxin, but the unique use of anti-CPA toxin antibodies is inadequate for the complete neutralization of *C. perfringens* type A-associated enteric disorders. Therefore, only an ideal multivalent vaccine comprising the recombinant C-terminal domain of CPA toxin, other toxins, and additional virulence factors might be efficacious for protecting against type A toxaemia.

## Conclusion

Collectively, in the present study, we first generated a recombinant baculovirus that expressed the C-terminal region of the CPA toxin of *Clostridium perfringens*. In the baculovirus, a *L21* leader sequence was inserted to enhance the expression of recombinant proteins. The rBaccCPA250–363H6 His-tagged protein was efficiently purified and it was confirmed that its presence did not result in any cytotoxicity and biological activity in the CaCo-2 cells and EYDT test, respectively. The specific polyclonal and monoclonal antibodies that were developed recognized the truncated recombinant CPA toxin, full length PLC, and native CPA in the Western blot and preliminary ELISA test. Further studies will be conducted for optimizing their use in immune-enzymatic assays. MAbs and antisera showed the ability to neutralize the lecithinase activity of the PLC toxin. Finally, the availability of these biologicals could contribute to the development of diagnostic assays and/or new recombinant protein vaccines.

## Data Availability

The data and the materials produced are available from the corresponding author after reasonable request and signing a material transfer agreement.

## Abbreviations

aa: Amino acids
CPA: *C. perfringens* alpha toxin
DTT: Dithiothreitol
ELISA: Enzyme-linked Immunosorbent Assay
EYDT: Egg yolk diffusion test
FBS: Foetal bovine serum
MAb: Monoclonal antibody
MW: Molecular weight
ON: Overnight
PCR: Polymerase chain reaction
PLC: Phospholipase C
rBacCPA250–363H6: Deleted/truncated recombinant C-terminal 6xHis-tagged CPA toxin produced in baculovirus
RT: Room temperature
SDS-PAGE: Sodium dodecyl sulphate–polyacrylamide gel electhrophoresis
